# Visual learning in a virtual reality environment upregulates immediate early gene expression in the mushroom bodies of honey bees

**DOI:** 10.1038/s42003-022-03075-8

**Published:** 2022-02-14

**Authors:** Haiyang Geng, Gregory Lafon, Aurore Avarguès-Weber, Alexis Buatois, Isabelle Massou, Martin Giurfa

**Affiliations:** 1 Research Centre on Animal Cognition, Center for Integrative Biology, CNRS, University of Toulouse, 118 route de Narbonne, F-31062, Toulouse, cedex 09 France; 2 College of Animal Sciences (College of Bee Science), Fujian Agriculture and Forestry University, 350002 Fuzhou, China; 3 Institut Universitaire de France (IUF), Paris, France; 4 Present Address: Institute of Neuroscience and Physiology, Department of Neurochemistry and Psychiatry, University of Gothenburg, Su Sahlgrenska, 41345 Göteborg Sweden

**Keywords:** Learning and memory, Visual system

## Abstract

Free-flying bees learn efficiently to solve numerous visual tasks. Yet, the neural underpinnings of this capacity remain unexplored. We used a 3D virtual reality (VR) environment to study visual learning and determine if it leads to changes in immediate early gene (IEG) expression in specific areas of the bee brain. We focused on *kakusei, Hr38* and *Egr1*, three IEGs that have been related to bee foraging and orientation, and compared their relative expression in the calyces of the mushroom bodies, the optic lobes and the rest of the brain after color discrimination learning. Bees learned to discriminate virtual stimuli displaying different colors and retained the information learned. Successful learners exhibited *Egr1* upregulation only in the calyces of the mushroom bodies, thus uncovering a privileged involvement of these brain regions in associative color learning and the usefulness of *Egr1* as a marker of neural activity induced by this phenomenon.

## Abstract (web summary)

The neural bases of visual learning in bees have been understudied, relative to the olfactory learning process. Using a 3D virtual reality environment and gene expression analyses, the neural underpinnings of visual learning are explored here.

## Introduction

Invertebrate models of learning and memory have proved to be extremely influential to determine where and when such experience-dependent plasticity occurs in the nervous system^1–6^. One of these models is the domestic honey bee *Apis mellifera*, which has been intensively investigated for its visual and olfactory-learning capacities^5,7,8^. Yet, the knowledge gained on the mechanisms of these abilities is disparate. While an extensive body of research has accumulated on the neural bases of olfactory learning and memory in bees^9^, practically nothing is known about the neural and molecular underpinnings of their visual learning and memory^10,11^. This asymmetry is due to the fact that olfactory-learning protocols use harnessed bees that learn to extend their proboscis to an odorant that has been forward-paired with sucrose water, while visual learning protocols use free-flying bees trained to choose a visual target where they collect sucrose reward^5,10^. Whilst the harnessing situation of olfactory-learning protocols facilitates the use of invasive techniques to record neural activity, the use of bees that commute freely between the hive and the experimental site precludes equivalent access to visual neural circuits.

Virtual-reality (VR) environments constitute a valuable tool to overcome this limitation. In such environments, tethered bees walking stationary on a treadmill are exposed to a controlled visual environment that allows studying decision making based on visual cues^12–17^. Under these conditions, bees learn and memorize simple and higher-order visual discrimination problems, which enables coupling the study of this visual learning with mechanistic analyses of brain activity^16,17^. VR setups may differ according to the degree of variation introduced by the bee movement into the visual environment. In closed-loop conditions, this variation is contingent on the movements of a tethered bee, thus creating a more immersive environment. In prior works, we introduced a 2D VR environment in which a tethered bee could displace laterally (from left to right and vice versa) a color stimulus on a frontal screen according to its association with sucrose reward of the absence of reward^12,14,18^. Here we moved towards a more realistic 3D VR environment which allowed, in addition, for stimulus expansions and retractions depending on forward or backward movements, respectively. In this arena, bees may therefore learn to discriminate colors but can also explore in a less restricted way the virtual world proposed to them.

One way to detect brain regions and pathways activated in this scenario is the quantification of immediate early genes (IEGs) in neural tissues^19^. IEGs are transcribed transiently and rapidly in response to specific stimulations inducing neural activity without *de novo* protein synthesis^20^. In mammals, IEGs, such as *c-fos*, *zif268*, and *Arc*, are regularly used as markers of neural activity during learning, memory and other forms of cellular plasticity such as long-term potentiation^21–23^. In insects, the use of IEGs as neural markers is less expanded as the number of candidate genes serving this goal is still reduced and the reliable detection of their expression is sometimes difficult^24^. Three of the IEGs reported for the honey bee are interesting as they have been related to a foraging context in which learning plays a fundamental role. The first one, termed *kakusei* (which means ‘awakening’ in Japanese), is a nuclear noncoding RNA transiently and strongly induced in the brain of European workers by seizures that can be induced by awakening them from anesthesia^25^. It is also activated after the experience of dancing in the hive following a foraging flight and in pollen foragers so that it seems related to the neural excitation resulting from foraging activities^26^. This IEG is activated within a subtype of Kenyon cells, the constitutive neurons of the mushroom bodies, which are a higher-order center in the insect brain^27^. A second IEG is the hormone-receptor 38 gene (*Hr38*), which is a transcription factor conserved among insects and other species including humans^28^, and which has been indirectly related to learning and memory in honey bees and other insects^29,30^. *Hr38* is also upregulated by foraging experiences in honey bees^29^ and bumblebees^30^ and by orientation activities upon hive displacement^31^. The third gene is the early growth response gene-1 (*Egr1*), whose expression is induced in the brain of honey bees and bumblebees upon foraging^29,30^ and orientation flights^32^, and which seems to be controlled by circadian timing of foraging^33^. None of these IEGs have been studied so far in the context of associative learning and memory formation in the honey bee.

We thus focused on these IEGs to characterize neural activation induced by visual learning in the brain of bees under 3D VR conditions. Bees had to learn to discriminate a rewarded color from a punished color^34–37^ and should retain this information in a short-term retention test. Our goal was to determine if successful learning and retention activate specifically certain regions in the brain, in particular the mushroom bodies, whose importance for olfactory learning and memory has been repeatedly stressed^5,38^, yet with a dramatic lack of equivalent evidence in the visual domain. Our results show that successful learners exhibited *Egr1* upregulation only in the calyces of the mushroom bodies, thus uncovering a privileged involvement of these brain regions in associative color learning.

## Results

### Color learning under 3D VR conditions

**Fig. 1:**
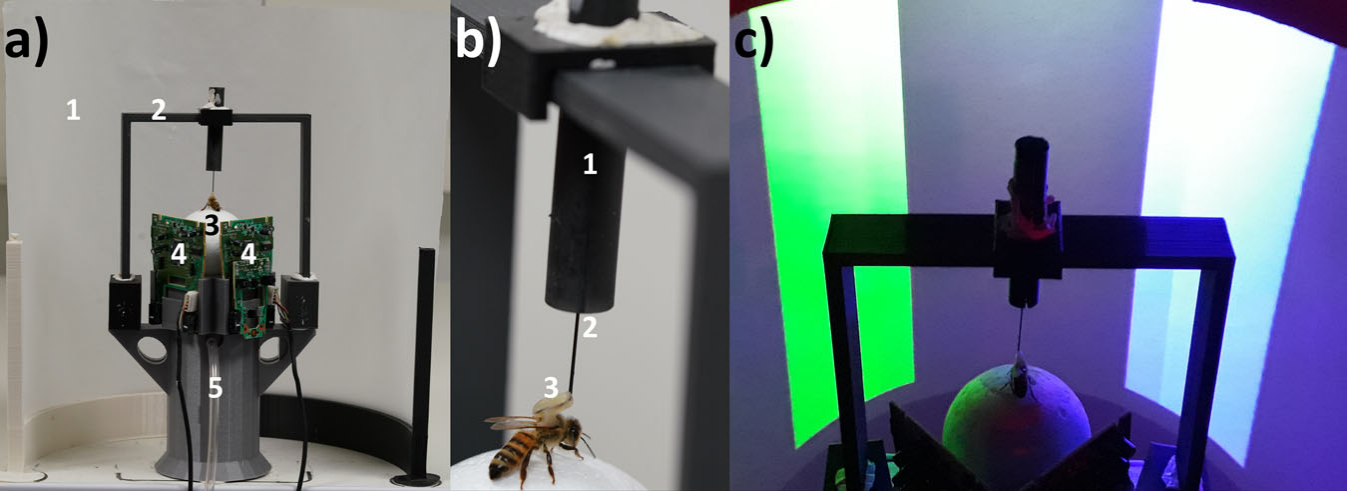
Experimental setup and 3D environment. **a** Global view of the VR system. (1) Semicircular projection screen made of tracing paper. (2) Holding frame to place the tethered bee on the treadmill. (3) The treadmill was a Styrofoam ball positioned within a cylindrical support (not visible) floating on an air cushion. (4) Infrared mouse optic sensors allowing to record the displacement of the ball and to reconstruct the bee’s trajectory. (5) Air arrival. The video projector displaying images (not visible) was behind the screen. **b** The tethering system. (1) Plastic cylinder held by the holding frame; the cylinder contained a glass cannula into which a steel needle was inserted. (2) The needle was attached to the thorax of the bee. (3) Its curved end was fixed to the thorax by means of melted bee wax. **c** Color discrimination learning in the VR setup. The bee had to learn to discriminate two vertical stimuli based on their different color and their association with reward and punishment. Stimuli were green and blue on a dark background. Color intensities were adjusted to avoid phototactic biases independent of learning.

Honey bee foragers were captured at an artificial feeder to which they were previously trained and brought to the laboratory where a tether was glued on their thorax. (Fig. 1a, b). They could be then attached to a holder that allowed adjusting their position on a treadmill, a polystyrene ball floating on a constant airflow produced by an air pump (see Methods for details). The VR setup consisted of this treadmill placed in front of a semi-cylindrical semi-transparent screen made of tracing paper (Fig. 1a). The movements of the walking bee on the treadmill were recorded by two infrared optic-mouse sensors placed on the ball support perpendicular to each other.

**Fig. 2:**
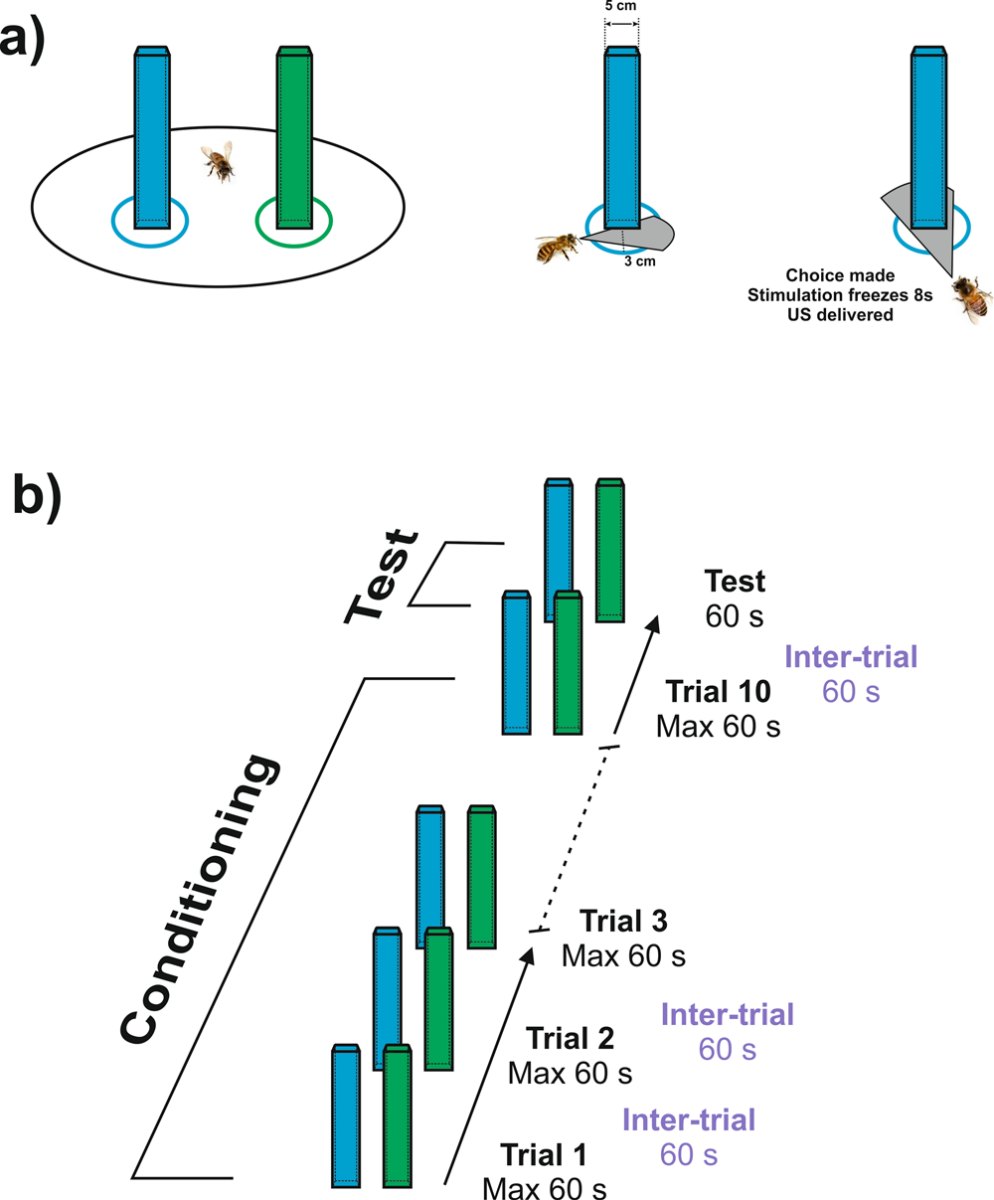
Choice criterion and conditioning protocol for color discrimination learning. **a** Choice criterion. Left: A bee facing the two virtual cuboids. Center: A bee approaching a target cuboid; the cuboid has not yet been centered by the bee (gray area). Right: A bee having centered the target cuboid (gray area). A choice was recorded when the bee reached an area of a radius of 3 cm centered on the cuboid and fixed it frontally. The cuboid image was then frozen during 8 s and the corresponding reinforcement (US) was delivered. **b** Conditioning protocol. Bees were trained along 10 conditioning trials that lasted a maximum of 1 min and that were spaced by 1 min (intertrial interval). After the end of conditioning, and following an additional interval of 1 min, bees were tested in extinction conditions with the two colored cuboids during 1 min.

**Fig. 3:**
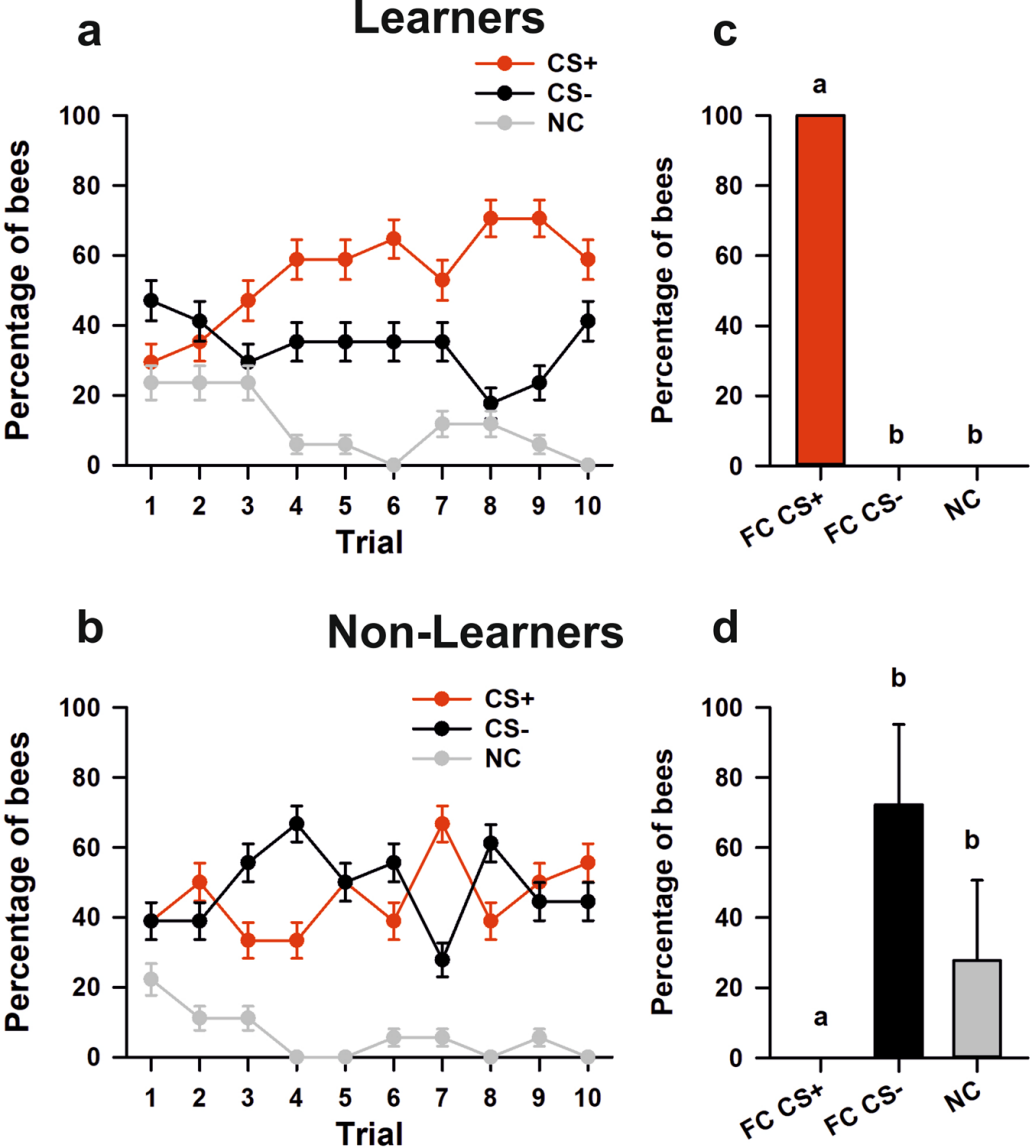
Discrimination learning in the VR setup. **a** Acquisition performance of learners (i.e., percentage of bees that chose the CS+ in the non-reinforced test; *n* = 17). The red, black, and gray curves show the percentages of bees choosing the CS+ , the CS− or not making a choice (NC), respectively. Bees learned the discrimination between CS+ and CS−. **b** Acquisition performance of non-learners (i.e., percentage of bees that chose the CS− or did not make a choice in the non-reinforced test; *n* = 18). These bees did not learn to discriminate the CS+ from the CS−. **c** Test performances of learners. Percentage of bees choosing in their first choice the CS+ (FC CS+), the CS− (FC CS−) or not making a choice (NC). Per definition, learners chose the CS+ in this test. Different letters on top of bars indicate significant differences (GLMM; *p* < 0.05). **d** Test performances of non-learners. Percentage of bees choosing in their first choice the CS+ (FC CS+), the CS− (FC CS−) or not making a choice (NC). Per definition, non-learners did not choose the CS+ . Different letters on top of bars indicate significant differences (GLMM; *p* < 0.05). In all panels, error bars indicate the 95% confidence interval.

Bees were trained to discriminate a green from a blue vertical cuboid against a black background during ten conditioning trials (Fig. 1c; see Supplementary Fig. 1 for color characteristics). Training consisted in pairing one of the cuboids (CS+) with a rewarding 1 M sucrose solution and the other (CS−) with an aversive 3 M NaCl solution^39,40^ (Fig. 2). Bees performed equally irrespective of the color trained (z = −0.97, *p* = 0.33). They were subdivided according to their test performance to distinguish those which showed successful discrimination (i.e., choice of the CS+ ; “*learners*”) from those which did not (“*non-learners*”). This distinction allowed subsequent brain gene analyses according to learning success. Bees that were unable to choose a stimulus in at least 5 trials were excluded from the analysis. Acquisition was significant for learners (*n* = 17) during conditioning trials (Fig. 3a; CS*Trial effect: χ^2^ = 33.68, df:2, *p* < 0.0001), confirming the occurrence of learning. Indeed, the percentages of bees responding to the CS+ and to the CS− differed significantly along trials (CS+ vs*.* CS*−*: CS*Trial; z = −5.46, *p* < 0.0001). Significant differences were also found when comparing the percentages of non-responding bees against the CS+ responding bees and against the CS− responding bees (NC vs. CS+: CS*Trial; z = 8.14, *p* < 0.0001; NC vs. CS−: CS*Trial; z = 4.59, *p* < 0.0001). Non-learners (*n* = 18) did also show a significant interaction (Fig. 3b; CS*Trial effect: χ^2^ = 7.66, df:2, *p* = 0.02), but this was introduced by the percentage of non-responding bees. These bees differed significantly along trials both from the bees responding to the CS+ (NC vs. CS+: CS*Trial; z = 6.10, *p* < 0.0001) and from the bees responding to the CS− (NC vs*.* CS−: CS*Trial; z = 6.07, *p* < 0.0001). On the contrary, the percentages of bees responding to the CS+ and to the CS− did not vary along with trials (CS+ vs. CS−: CS*Trial; z = −0.07, *p* = 1), consistently with the absence of learning.

**Fig. 4:**
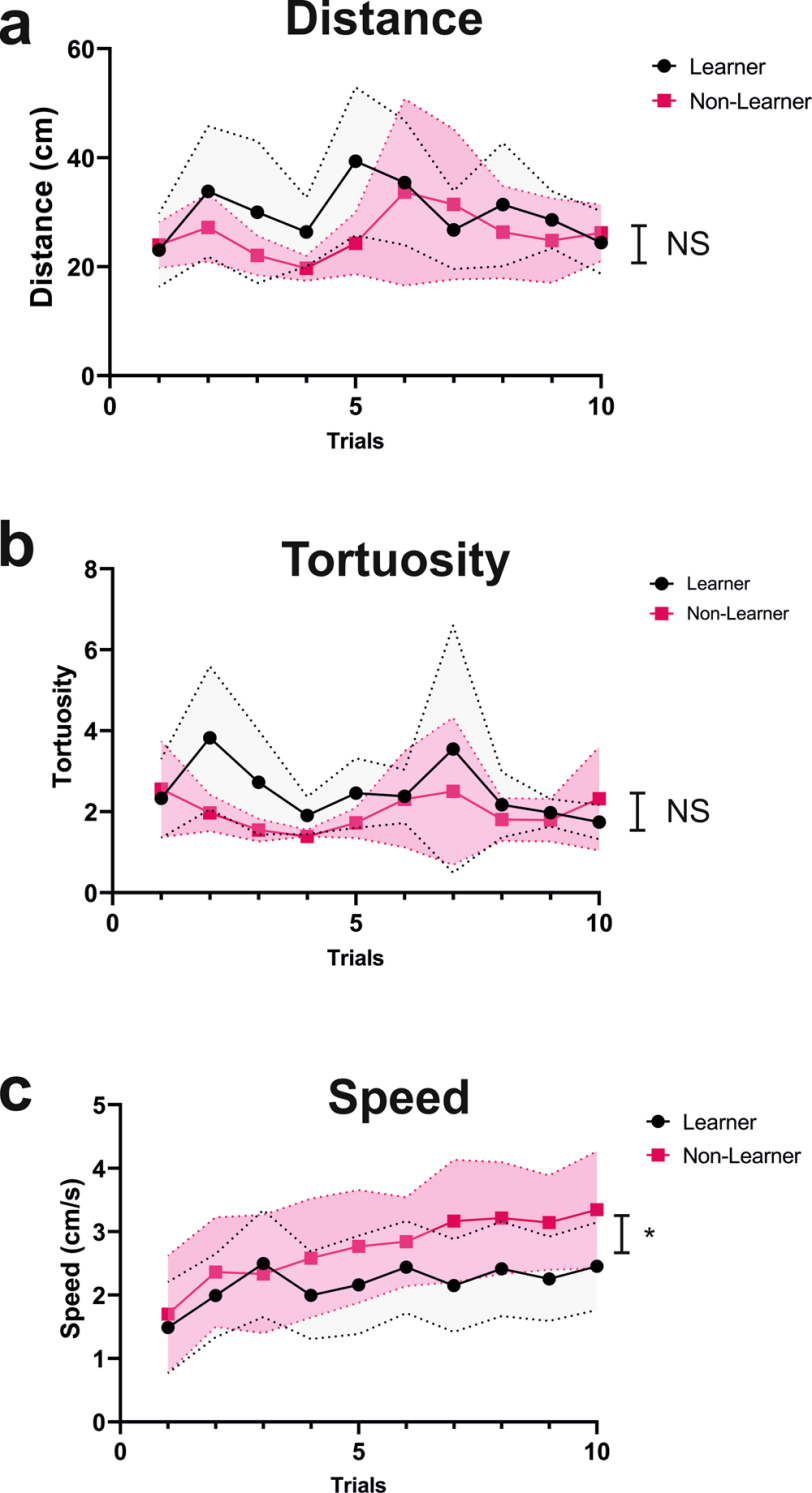
Motor components of learners (*n* = 17) and non-learners (*n* = 18) in the VR setup during conditioning. **a** Distance travelled (cm) during each conditioning trial. **b** Tortuosity of the trajectories (see text for explanation) during each conditioning trial. **c** Walking speed (cm/s) during each conditioning trial. The dashed lines above and below the curves represent the 95% confidence interval. Comparisons between curves refer to the significance of the interaction between the factors Trial (1–10) and Condition (learners vs. non-learners). All comparisons referring to Condition alone were non-significant. LMM; *p < 0.05; NS non-significant.

We next asked if differences between learners and non-learners could be due to differences in motor components. To answer this question, we analyzed for each conditioning trial the total distance walked, the walking speed, and the tortuosity of the trajectories. Tortuosity was calculated as the ratio between the total distance walked and the distance between the first and the last point of the trajectory connected by an imaginary straight line. When the ratio was 1, or close to 1, trajectories were straightforward while higher values corresponded to sinuous trajectories. The distance travelled (Fig. 4a) did neither vary along trials (Trial: χ^2^ = 0.24, df:1, *p* = 0.62) nor between learners and non-learners (Condition: χ^2^ = 1.10, df:1, *p* = 0.30; Condition*Trial: χ^2^ = 0.71, df:1, *p* = 0.40). Tortuosity (Fig. 4b) varied along trials (Trial: χ^2^ = 14.53, df:1, *p* < 0.001) but not between learners and non-learners (Condition: χ^2^ = 0.08, df:1, *p* = 0.80; Condition*Trial: χ^2^ = 0.42, df:1, *p* = 0.52). Finally, the walking speed (Fig. 4c) increased significantly along trials (Trial: χ^2^ = 30.49, df:1, *p* < 0.0001) but did not vary between learners and non-learners (Condition: χ^2^ = 1.43, df:1, *p* = 0.23); in this case, however, the interaction between Trial and Condition was significant (χ^2^ = 4.68, df:1, *p* < 0.05). This suggests that learners were slower than non-learners, which is reminiscent of a speed-accuracy trade-off reported in numerous experiments in bees^41–43^.

Finally, in the non-reinforced test, per definition learners (*n* = 17; Fig. 3c) chose correctly the CS+ (100% of the bees) while non-learners (*n* = 18; Fig. 3d) did either chose the CS− (72.22%) or did not perform any choice (27.78%). We thus focused on differences between learners and non-learners in the subsequent IEG analyses to uncover possible changes in neural activity induced by learning.

### IEG analyses in the honey bee brain following color learning under 3D VR conditions

**Fig. 5:**
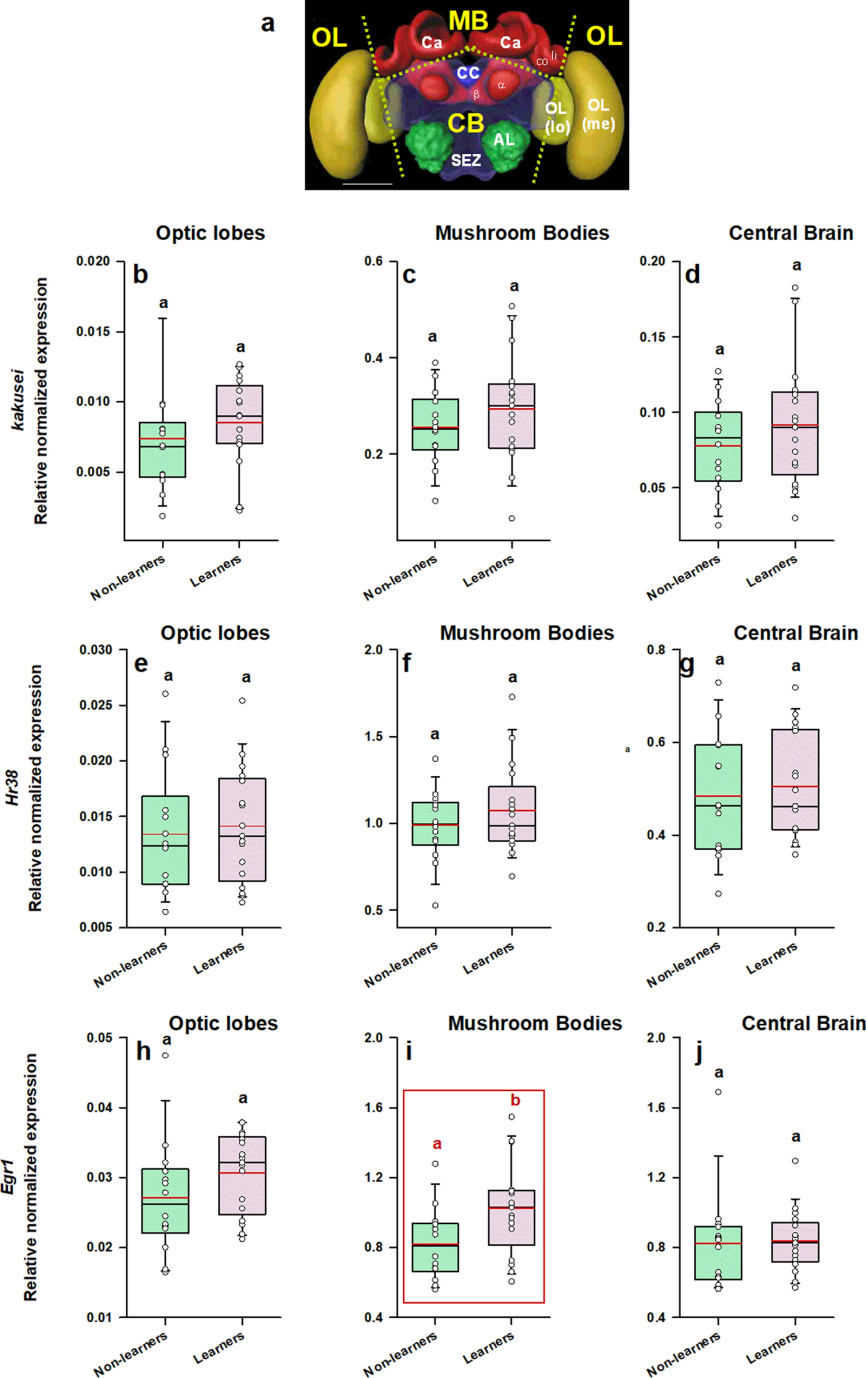
*Egr1*, but neither *kakusei* nor *Hr38*, shows significant variation of relative expression in the mushroom bodies following visual associative learning in a 3D VR environment. **a** Honey bee brain with sections used for quantifying IEG expression. Yellow labels indicate the brain regions used for the analysis: MB mushroom body, CB central brain, OL optic lobes. The dashed lines indicate the sections performed. Ca calyx of the mushroom body, li lip, co collar, α and β α and β lobes of the mushroom body, CC central complex, AL antennal lobe, SEZ subesophagic zone, OL optic lobe, Me medulla, lo lobula. **b**–**d** Relative normalized expression of *kakusei*, of *Hr38* (**e**–**g**) and of *Egr1* (**h**–**j**) in three main regions of the bee brain, the optic lobes (**b**, **e**, **h**), the calyces of the mushroom bodies (**c**, **f**, **i**) and the central brain (**d**, **g**, **j**). The expression of each IEG was normalized to the expression of two genes of reference (*Actin and Ef1α*) in the case of the MB and the CB, and of *Actin* alone in the case of the OL (see Supplementary Fig. 2). The range of ordinates was varied between target genes to facilitate appreciation of data scatter. IEG expression was analyzed in individual brains of bees belonging to two categories: *learners* (conditioned bees that responded correctly and chose the CS+ in their first choice during the non-reinforced test; *n* = 17) and non-learners (conditioned bees that did not choose the CS+ in their first choice during the non-reinforced test; *n* = 14). The range of ordinates was varied between target genes to facilitate appreciation of data scatter. Boxplots show the mean value in red. Error bars define the 10th and 90th percentiles. Red boxes indicate cases in which significant variations were detected. Different letters on top of boxplots indicate significate differences (two-sample *t*-test; *p* < 0.05).

We aimed at determining if visual learning in VR induces post-learning transcriptional changes, which might participate in amplifying neural activity reflecting associative color learning. To this end, we performed RT-qPCR in individual brains of learners and non-learners, which were collected 1 h after the retention test and placed in liquid nitrogen until brain dissection. We analyzed relative expression levels of *kakusei*, *Hr38* and *Egr1* (see Table 1) in three main brain regions^44^ (Fig. 5a): the optical lobes (OL), the upper part of the mushroom bodies (i.e., the mushroom-body calyces or MB Ca) and the remaining central brain (CB), which included mainly the central complex, the subesophageal zone and the peduncula and lobes (α and β lobes) of the mushroom bodies. Two reference genes were used for the normalization, *Ef1α* (E = 106%) and *Actin* (E = 110%), which proved to be the best choice for the normalization (see Table 1). The Cq values of these reference genes for the different conditions of this experiment are shown in Supplementary Fig. 2. Stability was granted for both genes and experimental groups (learners and non-learners) for the MB and the CB. In the case of the OL, *Ef1α* varied significantly between groups. Thus, normalization used the product of the two reference genes for MB and CB while only actin could be used to normalize OL data. No cross-comparisons between brain regions or genes were performed.

**Table 1:** Primer sequences used to quantify RNA expression of genes of interest and reference genes by RT-qPCR.

Type of gene	Target	Primer sequence 5’ →3’	Amplicon length (bp)	E (%)	R^2^
Target genes	*Kakusei*	CTACAACGTCCTCTTCGATT (forward) CCTACCTTGGTATTGCAGTT (reverse)	149	96.4	0.991
	*Hr38*	TGAGATCACCTGGTTGAAAG (forward) CGTAGCAGGATCAATTTCCA (reverse)	118	106	0.995
	*Egr1*	GAGAAACCGTTCTGCTGTGA (forward) GCTCTGAGGGTGATTTCTCG (reverse)	138	109	0.991
Reference genes	*Ef1α*	AAGAGCATCAAGAGCGGAGA (forward) CACTC TTAATGACGCCCACA (reverse)	148	106	0.993
	*Actin*	TGCCAACACTGTCCTTTCTG (forward) AGAATTGACCCACCAATCCA (reverse)	156	110	0.995

Amplicon length (bp), efficiency (E, %), and the coefficient of correlation obtained for the standard curve (R^2^) are also shown.

*Hr38* hormone-receptor 38 gene, *Egr1* early growth response gene-1, *Ef1α* elongation factor 1 α gene.

Figure 5b-d shows the relative normalized expression of *kakusei* for the three brain regions considered in the case of learners and non-learners. No significant variations of relative expression were found between these two groups for the three regions considered (two-sample *t*-test; Fig. 5b, OL: t_29_ = 0.83, *p* = 0.42; Fig. 5c, MB: t_29_ = 1.09, *p* = 0.29; Fig. 5d, CB: t_29_ = 1.04, *p* = 0.31). Thus, *kakusei* was unable to reveal learning-induced variations in neural activity under our experimental conditions. The normalized expression of *Hr38* (Fig. 5e-g) was also insufficient to uncover learning-related differences between learners and non-learners (Fig. 5e, OL: t_29_ = 0.37, *p* = 0.72; Fig. 5f, MB: t_29_ = 0.99, *p* = 0.33; Fig. 5g, CB: t_29_ = 0.44, *p* = 0.67). However, a significant upregulation of *Egr1* expression was found in the mushroom bodies of learners when compared to non-learners (Fig. 5i, t_29_ = 2.40, *p* = 0.02). Differences in *Egr1* expression between learners and non-learners were neither found in the OL (Fig. 5h, t_29_ = 1.48, *p* = 0.15) nor in the CB (Fig. 5j, t_29_ = 0.17, *p* = 0.86), thus showing that learning-dependent variation in IEG expression was circumscribed to the calyces of the mushroom bodies and that *Egr1* was more sensitive than both *Hr38* and *kakusei* to detect changes in neural activity induced by associative learning.

## Discussion

Our work shows that visual discrimination learning under virtual-reality conditions leads to an enhancement of IEG expression in the case of *Egr1* in the calyces of the mushroom bodies in successful honey bee learners. Learning success did not correlate with differences in distance travelled or tortuosity of trajectories, i.e., with differences in an exploratory drive (Fig. 4), but was correlated with differences in walking speed as learners tended to be slower than non-learners. Although strictly speaking the two categories did not differ with respect to this parameter, the significant interaction between Trial and Condition suggests a speed-accuracy trade-off in which individuals taking more time to decide can improve the accuracy of their decisions^41–43^. Differences in *Egr1* expression were thus related to learning success and not to differences in exploratory components. For the other two IEGs analyzed, *kakusei* and *Hr38*, no learning-dependent changes could be detected in the different brain regions considered, even if prior reports indicated similar levels of expression for the three IEGs in the brain of bees engaged in foraging^29,30,33,45^ and orienting around the hive^29–31^. Our work demonstrates therefore that this similarity does not necessarily reflect a relationship with associative learning and memory as only *Egr1* acted as a bona fide marker of learning success in the bee brain under our experimental conditions and revealed the implication of the calyces of the mushroom bodies in associative visual learning and memory in honey bees.

### Differential expression of IEGs in the honey bee brain as related to visual learning

*Kakusei* did not vary in the brain regions considered, under the experimental conditions defined in our work. This IEG does not have orthologous genes in other taxa and its role in honey bees is unclear. It is induced by seizures following anesthesia^25,27,45,46^ and thermal stimulation^46^, but also by foraging and reorientation activity following hive displacement^25,31,45^. These experiences increase *kakusei* expression in the mushroom bodies^25^ but also in the optic lobes^25,27,45^ and the dorsal lobe^27^. Our results suggest that its enhanced expression in foragers or in orienting bees is not necessarily related to the learning occurring in these contexts.

Differential expression of *kakusei* with respect to an inducing treatment (typically, an induced seizure) starts around 15 min post treatment^25,31,46^ but continues during longer periods which may go beyond 60 min^46^. Thus, the waiting time of 60 min between test and brain freezing in our experiments was appropriate to detect changes in *kakusei* as a result of associative visual learning. However, as other temporal analyses of *kakusei* expression reported decay in expression beyond 30 min^25^, the possibility that our sampling period was too long to capture changes in *kakusei* expression cannot be excluded.

This concern does not apply to *Hr38* and *Egr1*, for which temporal expression analyses showed a systematical increase at the time chosen for our experiments^30^. As in the case of *kakusei*, no learning-related changes were detected in *Hr38* expression across the brain regions considered. This hormone-receptor gene has been indirectly related to learning and memory in honey bees and other insects^29,30^ and is also upregulated by foraging experiences in honey bees^29^ and bumblebees^30^ and by orientation activities upon hive displacement^31^. Despite its involvement in these activities, it did not reveal learning-dependent changes in neural activity in the experimental context defined by our setup and training protocol.

Only *Egr1* reported a significant variation in the mushroom-body calyces of learners in relation to non-learners (Fig. 5). As for the two other IEGs, the expression of this early growth response gene is enhanced in the brain of honey bees and bumblebees upon foraging^29,30^ and orientation flights^32^. Yet, in this case, *Egr1* was sensitive enough to report differences in neural activity related to learning success in our experimental conditions. Learners and non-learners were identical in their experience and handling all along with the experiment and they only differed in learning success. Thus, differences in *Egr1* expression demonstrate that associative color learning is accompanied by increased neural activity in the calyces of the mushroom bodies.

### The role of mushroom bodies for visual learning and memory

Although the crucial role of mushroom bodies for the acquisition, storage and retrieval of olfactory memories has been extensively documented in bees^7,38,47^ and other insect species^2,3,48^, less is known about their implication in visual learning and memory. In the honey bee, the fact that visual learning was mainly studied using free-flying bees trained to choose visual targets precluded its study at the cellular level^13^. The neural circuits for color processing are known in the bee brain^49–52^ but evidence about plasticity-dependent changes in these circuits remains scarce. Such changes could occur at multiple stages, as is the case in olfactory circuits mediating olfactory learning^9^. Upstream the mushroom bodies, inner-layer lobula and inner medulla neurons project to both the mushroom bodies and the lateral protocerebrum^49,50,53^ and exhibit color sensitivity, color opponency and temporally complex patterns including adaptation and entrainment^49,53,54^. These patterns are important for color coding and discrimination and could be subjected to experience-dependent changes in activity^55^.

The implication of mushroom bodies in visual learning and memory in the bee is expected given the parallels between visual and olfactory inputs at the level of the calyces. While afferent projection neurons convey olfactory information to a subdivision of the calyces, the lip^56^, afferent neurons from the lobula and the medulla, which are part of the optic lobes, convey visual information to other calyx subdivisions, the collar and the basal ring^50,57^. In spite of this similarity, studies addressing the role of mushroom bodies in honey bee visual learning and memory remain rare. The recent development of protocols for the study of *aversive* visual learning (association between a color light and an electric shock delivered to walking bees enclosed in a box compartment)^44,58^ has shown the possible implication of mushroom bodies in this form of learning. In a pharmacological study, in which one half of a chamber was illuminated with one color and paired with shock while the other half was illuminated with a different color not paired with shock, bees learned to escape the shock-paired light and spent more time in the safe light after a few trials^59^. When ventral lobe neurons of the mushroom bodies were silenced by procaine injection, bees were no longer able to associate one light with shock. By contrast, silencing one collar region of the mushroom-body calyx did not alter behavior in comparison with that of controls^59^. The latter result does not exclude a role for the calyces in visual learning, as blocking one of four collar regions may not have a significant impact on learning. In a different study, bees were trained to inhibit their spontaneous phototaxis by pairing the attracting light with an electric shock^44^. In this case, learning induced an increase in the dopaminergic receptor gene *Amdop1* in the calyces of the mushroom bodies, consistently with the role of dopaminergic signaling for electric-shock representation in the bee brain^60,61^.

In the fruit fly, the study of the role of mushroom bodies for visual learning and memory has yielded contradictory results. Flies suspended within a flight simulator learn to fly towards unpunished visual landmarks to avoid heat punishment delivered to their thorax; mushroom-body deficits do not affect learning so these structures were considered dispensable for visual learning and memory^62^. Similarly, learning to discriminate colors in a cylindrical container made of a blue-lit and a yellow-lit compartment, one of which was associated with aversive shaking, was not affected in mushroom-body mutants^63^. Visual place learning by flies walking within a cylindrical arena displaying landmarks can also take place in the absence of functional mushroom bodies but requires the central complex^64^. Yet, the dispensability of mushroom bodies for visual learning and memory in fruit flies has been questioned by experiments in which appetitive and aversive color learning and discrimination were studied in an arena in which blue and green colors were presented from below. Walking flies learned both the appetitive (based on pairing one color with sugar) and the aversive discrimination (based on pairing one color with electric shock) but failed if mushroom-body function was blocked using neurogenetic tools^65^. Thus, the role of mushroom bodies for visual learning and memory in fruit flies may be both task- and learning-specific. In addition, the dominance of olfactory inputs to the mushroom bodies may overshadow their role for visual learning in *Drosophila*.

### IEG expression within the mushroom bodies in relation to visual learning

Kenyon cells are the constitutive neurons of mushroom bodies. Their somata are located both within the mushroom-body calyces and adjacent to them. Thus, our brain sectioning (see Fig. 4a) collected them massively. Detecting IEG activation in the mushroom bodies upon visual learning may be particularly difficult as learning-dependent changes in neural activity may be subtle due to the characteristic sparse neural activity observed at the level of the calyces. This reduced activity, which has been revealed in studies on olfactory coding^66–68^ and odor-related learning^69^, can also be a hallmark of visual processing and visual learning. Sparse neural coding of odorants is in part due to GABAergic inhibition by feedback extrinsic mushroom-body neurons acting on Kenyon cells^70,71^, the constitutive neurons of the mushroom bodies. These GABAergic neurons, present both in bees and flies^70,72,73^, suppress Kenyon-cell activity to maintain sparse, neural coding, and may render it difficult to detect variations of IEG expression in the calyces. Yet, we were able to find differences that were dependent on the experience of the animals analyzed. Such differences might vary according to the difficulty of the learning problem considered. For instance, higher GABAergic input is required in the calyces to solve non-linear discriminations, in which subjects have to inhibit response summation to the simultaneous presentation of stimuli A and B, which are rewarded when presented alone but non-rewarded when presented together. Bees that learn to solve this discrimination in the olfactory domain require inhibitory GABAergic feedback in the calyces to this end^47^. Such a requirement could translate into a different form of IEG expression in this brain region as a consequence of a more complex discrimination learning.

Recent work on gene expression in the Kenyon cells of honey bees revealed the existence of various cell subtypes/populations with unique gene-expression profiles and cell body morphology^74^. Among these populations, small Kenyon cells (sKC)^75^, formerly called inner Kenyon cells^76^, are found in the central, inner core of the MB calyces and express preferentially three genes, *EcR*, *E74*, and *Hr38*, the latter being higher in the brain of foragers than in nurses^74^. Unfortunately, no information on *Egr1* was reported in this analysis. Yet, another study that did not distinguish between Kenyon-cell subtypes reported that the expression of *Egr1* is enriched in Kenyon cells compared to the rest of the brain^32^ and that this enrichment might be related to learning and memory given its association with the orientation flights of bees^32^ and with foraging activities^29,30,33^. However, the sensory cues and behavioral programs participating in both foraging and orientation are multiple so it is difficult to sustain such a claim in the absence of a controlled learning experiment. For instance, *Egr1* is also upregulated in the brain of honey bees upon seizure induction^77^, with no relation to foraging or orientation. Only specific experiments like the one performed in this work can reveal whether increases in this and other IEGs reflect neural activity induced by associative learning.

Consistently with the notion that sKCs may be particularly relevant for learning and memory formation, phosphorylated (activated) cAMP-response element-binding protein (pCREB) is enriched in these sKCs in the honey bee^78^. CREB is a nuclear protein that modulates the transcription of genes required for the cellular events underlying long-term memory (LTM) formation in both invertebrates and vertebrates^79–82^ and its activation leads also to the expression of IEGs. It is thus possible that the increased expression of *Egr1* induced by visual learning and memory formation is localized within sKCs, and that this increase results from CREB activation. In our experiments, the reinforced tests were done shortly after the last conditioning trial and only one hour elapsed since the end of the test and the collection of brains for IEG analysis (a time necessary for the expression of the IEGs selected). This period does not correspond with the temporal requirements for olfactory LTM formation in the standard view of memory dynamics in the honey bee, where a protein-synthesis-ependent LTM is expected after 24 h post-conditioning^83^. However, recent work on olfactory memory formation has shown that protein-synthesis-dependent memories arise much earlier and with fewer conditioning trials than previously thought^84^. Whether our visual conditioning leads to protein-synthesis-dependent LTM, mediated by CREB activation, remains to be determined.

Taken together, our results show both the implication of mushroom bodies in appetitive visual learning in honey bees and the usefulness of *Egr1* as a marker of neural activity induced by these phenomena under our experimental conditions. The learning success in our VR setup was 50%, which contrasts with the higher learning rates observable for similar color discriminations in the case of free-flying bees. This decrease may be due to several reasons such as the impossibility to return to the hive between rewarded experiences, the tethering conditions and the resulting reduction in active vision. As the tethering impedes, in part, free movements, it may affect the possibility of actively scanning the images perceived, impairing thereby the possibility of extracting target information and learning. In spite of these restrictions, our setup allowed us to segregate between learners and non-learners and achieve relevant analyses to answer questions on the neural and molecular underpinnings of associative visual learning. It constitutes therefore a valuable tool for further studies on the mechanisms of visual cognition in bees.

The protocol used to train the bees in our work consisted of elemental discrimination between a rewarded and non-rewarded color. Yet, bees are well known for remarkable visual performances, which include the non-elemental learning of concepts and relational rules^85–87^. It is, therefore, possible that different forms of learning, which recruit different brain regions^47^, may reveal experience-dependent neural activation through different IEGs and with different temporal dynamics. Moreover, IEG upregulation may not always be the hallmark of successful learning as in some cases inhibition of neural activity may be crucial for plastic changes in behavior. Thus, addressing if IEG expression varies qualitatively and quantitatively according to learning type and complexity is of fundamental importance. Furthermore, including different intervals post-conditioning is important to characterize possible activity changes related to the formation of different memory phases in different regions of the bee brain. Last, but not least, our results highlight the value of virtual-reality conditions for further explorations of the neural and molecular underpinnings of visual learning and memory in bees.

## Methods

Honey bee foragers (*Apis mellifera*) were obtained from colonies located in our apiary at the University Paul Sabatier. Only foragers caught upon landing on a gravity feeder filled with a 0.9 M sucrose solution were used in our experiments to ensure high appetitive motivation. Captured bees were brought to the laboratory where they were placed on ice for five minutes to anesthetize them and facilitate the fixation of a tether glued to their thorax by means of melted wax (Fig. 1a). After being attached to the tether, each bee was placed on a small (49 mm diameter) Styrofoam ball for familiarization with the treadmill situation. Bees were provided with 5 μl of 1.5 M sucrose solution and kept for 3 h in this provisory setup in the dark. They were then moved to the VR arena and used for the experiments.

Once in the VR setup, the bee was attached to a holder that allowed adjusting its position on the treadmill (Fig. 1b), a polystyrene ball (diameter: 5 cm, weight: 1.07 g) held by 3D-printed support and floating on a constant airflow produced by an air pump (airflow: 555 ml/s; Aqua Oxy CWS 2000, Oase, Wasquehal, France).

### VR setup

The VR setup consisted of the treadmill and of a half-cylindrical vertical screen made of semi-transparent tracing paper, which allowed the presentation of a 180° visual environment to the bee (diameter: 268 mm, height: 200 mm, distance to the bee: 9 cm Fig. 1a, b) and which was placed in front of the treadmill. The visual environment was projected from behind the screen using a video projector connected to a laptop (Fig. 1a). The video projector was an Acer K135 (Lamp: LED, Definition: 1280 × 800, Brightness: 600 lumens, Contrast ratio: 10,000:1, Minimum Vertical Sync: 50 Hz, Maximum Vertical Sync: 120 Hz, Minimum Horizontal Sync: 30.10^3^ Hz, Maximum Horizontal Sync: 100.10^3^ Hz)^14^. The movements of the walking bee on the treadmill were recorded by two infrared optic-mouse sensors (Logitech M500, 1000 dpi, Logitech, Lausanne, Switzerland) placed on the ball support perpendicular to each other.

Experiments were conducted under 3D closed-loop conditions, i.e., rotations of the ball displaced the visual stimuli not only laterally but also towards the bee. To generate these conditions, we developed a custom software by means of the Unity engine (version 2018.3.11f1). The open-source code is available at https://github.com/G-Lafon/BeeVR. The software updated the position of the bee within the VR every 0.017 s. A displacement of 1 cm on the ball corresponds to an equivalent displacement in the VR landscape. Moving 1 cm on the ball towards an object increased the visual angle of the object by ca. 1.7°. Based on the ball movements, our software calculated the position of the walking bee and its heading, and determined which object was centered on the screen.

### Visual stimuli

Bees had to discriminate two vertical cuboids (Fig. 1c) based on their different colors and association with reward and punishment. The colors of the cuboids (see Supplementary Fig. 1) were blue (RGB: 0, 0, 255, with a dominant wavelength of 450 nm and an irradiance of 161,000 μW) and green (RGB: 0, 100, 0, with a dominant wavelength of 530 nm and an irradiance of 24,370 μW/cm^2^). They were displayed on a black background (RGB: 0, 0, 0). These colors were chosen based on previous work showing their successful learning in the VR setup^14^.

Each cuboid had a 4.5 × 4.5 cm base when projected onto the screen and occupied the entire vertical extent of the screen irrespective of the bee’s position. The visual angle subtended by each cuboid to the bee’s eye was 28°, which ensured that choices were guided by the color properties of the stimuli^88^. The cuboids were positioned at −50° and +50° from the bee’s body axis at the beginning of each trial. Approaching a cuboid within an area of 3 cm surrounding its virtual surface followed by direct fixation of its center was recorded as a choice (Fig. 2a).

### Conditioning and testing at the treadmill

Bees were trained using differential conditioning, which promotes better learning performances owing to the presence of penalized incorrect color choices that results in an enhancement of visual attention^36^.

Bees were trained during 10 consecutive trials using a differential conditioning procedure (Fig. 2b) in which one of the cuboids (i.e., one of the two colors, green or blue) was rewarded with 1.5 M sucrose solution (the appetitive conditioned stimulus or CS+) while the other cuboid displaying the alternative color (the aversive conditioned stimulus or CS−) was associated with 3 M NaCl solution. The latter was used to increase the penalty of incorrect choices^39,40,89,90^. To avoid directional biases, the rewarded and the punished color cuboids were swapped between the left and the right side of the virtual arena in a pseudo-random manner along with trials. Moreover, a reconstruction of the trajectories of the bees analyzed did not show side biases.

A dark screen was shown initially to the bees. During training trials, each bee faced the two cuboids. The bee had to choose the CS+ cuboid by walking towards it and centering it on the screen. Colors were equally and randomly assigned to the CS+ and the CS− category during training. If the bee reached the CS+ within an area of 3 cm in the virtual environment (i.e., if the cuboid chosen by the bee subtended 53° in its horizontal extent) and centered it, the screen was locked during 8 s to ensure fixation. This allowed the delivery of sucrose solution in case of a correct choice, or of NaCl in case of an incorrect choice. Solutions were delivered for 3 s by the experimenter who sat behind the bee and used a toothpick to this end. The toothpick touched first the antennae and then the mouthparts during the 8 s in which the screen was locked on the cuboid fixated by the bee. Each training trial lasted until the bee chose one of both stimuli or for a maximum of 60 s (no choice). Trials were separated by an intertrial interval of 60 s during which the dark screen was presented. Bees that were unable to choose a stimulus (i.e., that did not fulfill the criterion of a choice defined above) in at least 5 trials were excluded from the analysis. From 216 bees trained, 75 were kept for analysis (~35%).

After the last training trial, each bee was subjected to a non-reinforced test that lasted 60 s (Fig. 2b). Test performance allowed distinguishing learners (i.e., bees that chose the CS+ as their first choice in the test) from non-learners (i.e., bees that either chose the CS− in their first test choice or that did not make any choice during the test). IEG expression was compared between these two groups, which had the same sensory experience in the VR setup and which differed only in their learning success.

### Brain dissection

One hour after the test, bees were decapitated, and the head was instantly frozen in a nitrogen solution. The period between post-test and brain collection was chosen to allow induction of the three IEGS studied (typically, 15 or more min in the case of kakusei^25,46^ and 30–60 min in the case of *Hr38*^*31*^ *and Egr1*^*30*^). The frozen bee head was dissected on dry ice under a microscope. First, the antennae were removed and a window was cut in the upper part of the head capsule, removing the cuticle between the compound eyes and the ocelli. Second, the glands and tracheae around the brain were removed. Third, the retinas of the compound eyes were also removed.

The frozen brain was cut into three main parts for IEG analyses (Fig. 4a): the optic lobes (OL), the upper part of the mushroom bodies (the mushroom-body calyces, MB Ca) and the remaining central brain (CB), which included mainly the central complex (CC), the subesophageal zone (SEZ) and the peduncula of the mushroom bodies (α and β lobes). Samples were stored at −80 °C before RNA extraction. During the dissection process, one of these three regions was lost in 4 non-learners brains As only bees for which all regions were available were kept in the analyses, the sample sizes of the non-learners differ between the behavioral (*n* = 18) and the molecular analyses (*n* = 14).

### RNA extraction and reverse transcription

The RNAs from the three sections mentioned above (OL, MB Ca, and CB) were extracted and purified using the RNeasy Micro Kit (Qiagen). The final RNA concentration obtained was measured by spectrophotometry (NanoDrop™ One, Thermo Scientific). A volume of 10 µl containing 100 ng of the RNA obtained was used for reverse transcription following the procedure recommended in the Maxima H Minus First Strand cDNA Synthesis kit (Thermoscientific, 0.25 µl of random hexamer primer, 1 µl of 10 mM dNTP mix, 3.75 µl of nuclease-free H_2_O, 4 µl 5× RT Buffer, and 1 µl Maxima H Minus Enzyme Mix).

### Quantitative polymerase chain reaction (RT-qPCR)

All the primers used for target and reference genes generated amplification products of ~150 pb. The efficiencies of all reactions with the different primers used were between 95 and 110 % (Table 1). Their specificity was verified by analyzing the melting curves of the qRT-PCR products (see Supplementary Fig. 2). Two reference genes (*Ef1α* and *Actin*) were used for normalization.

Expression was quantified using a SYBR Green real-time PCR method. Real-time PCR was carried out in 384-Well PCR Plates (Bio-Rad) covered with Microseal ‘B’ PCR Plate Sealing Film (Bio-Rad). The PCR reactions were performed using the SsoAdvancedTM Universal SYBR® Green Supermix (Bio-Rad) in a final volume of 10 μl containing 5 μl of 2× SsoAdvancedTM Universal SYBR® Green Supermix, 2 μl of cDNA template (1:3 dilution from the reverse transcription reaction), 0.5 μl of 10 μmol of each primer and 2 μl of ultrapure water. The reaction conditions were as follows: 95 °C for 30 s followed by 40 cycles of 95 °C for 10 s, 55 °C for 30 s, and a final step at 95 °C for 10 s followed by a melt curve from 55 °C to 95 °C with 0.5 °C per second. The reaction was performed in a CFX384 Touch Real-Time PCR Detection System (Bio-Rad) and analyzed with the software Bio-Rad CFX Manager.

Each sample was run in triplicates. If the triplicates showed too much variability (SD > 0.3), the furthest triplicate was discarded. If the two remaining triplicates still showed too much variability (SD > 0.3) the sample was discarded. The samples were subjected to relative quantification and normalization. First, for each sample and for each reference gene per brain region, the relative quantity (Qr) was computed using the difference between the mean Ct value of each sample and the highest mean Ct value (ΔCt), using the following formula: Qr = (1 + E)^ΔCt^ (with E = efficiency of the reaction). Then a normalization factor for each sample was obtained computing the geometric mean of the relative quantities obtained for the reference genes in the corresponding samples (ΔΔCt).

### Statistics and reproducibility

#### Behavioral data

The first choice of the bees was recorded during the conditioning trials and the non-reinforced test. In this way, we established for each trial and test the percentages of bees choosing first each of the stimuli displayed or not choosing a stimulus (±95% confidence interval).

Test percentages were analyzed within groups by means of a generalized linear mixed model (GLMM) for a binomial family in which the individual identity (Bee) was considered as a random factor (individual effect) while the choice category (CS+ , CS−, NC) was fitted as a fixed effect; z values with corresponding degrees of freedom are reported throughout for this kind of analysis.

For each acquisition trial, we recorded motor variables such as the total distance walked, the walking speed, and the tortuosity of the trajectories^91^. Tortuosity was calculated as the ratio between the total distance walked and the distance between the first and the last point of the trajectory connected by an imaginary straight line. When the ratio was 1, or close to 1, trajectories were straightforward while higher values corresponded to sinuous trajectories^91^. The analysis of these continuous variables was done using a linear mixed model (lmer function) in which the individual identity (*Bee ID*) was a random factor and the experimental condition (Condition) and trial number (Trial) were fixed factors^91^. Statistical analyses were performed using R 3.5.1^92^. The package lme4 was used for GLMMs and LMMs.

#### Gene-expression data

Statistical differences in gene expression were assessed for reference genes to check for stability and for target genes within a given brain region using One-Factor ANOVA for independent groups in the case of multiple comparisons or two-sample *t*-test in the case of dual comparisons. Pots hoc comparisons between groups were performed by means of a Tukey test following ANOVA. No cross-comparisons between brain regions or genes were performed due to within-area normalization procedures. Statistical analyses were done either with R 3.5.1 software^92^ or with Statistica 13 Software (TIBCO® Data Science).

### Reporting summary

Further information on research design is available in the Nature Research Reporting Summary linked to this article.

## Supplementary information

Supplementary Materials

Reporting Summary

## Data Availability

The datasets generated during this study are available at figshare.com with the following accession ID: 10.6084/m9.figshare.14994363.v1.

## Code availability

This work generated no code.
