# Supplementary material for: Visual learning in a virtual reality environment upregulates immediate early gene expression in the mushroom bodies of honey bees
Source: Commun Biol. 2022 Feb 14;5:130. doi: 10.1038/s42003-022-03075-8 (PMC8844430; doi:10.1038/s42003-022-03075-8)
Supplement: Supplementary file 1 — Supplementary Materials [file 42003_2022_3075_MOESM1_ESM.pdf]

Supplementary Information

Visual learning in a virtual reality environment upregulates immediate early gene expression in the mushroom bodies of honey bees

Haiyang Geng*[1,2], Gregory Lafon[1]*, Aurore Avarguès-Weber[1], Alexis Buatois[1,¥,§], Isabelle Massou[1,§], Martin Giurfa[1,2,3§]

[1] Research Centre on Animal Cognition, Center for Integrative Biology, CNRS, University of Toulouse, 118 route de Narbonne, F-31062 Toulouse cedex 09, France.

[2] College of Animal Sciences (College of Bee Science), Fujian Agriculture and Forestry University, Fuzhou 350002, China.

[3] Institut Universitaire de France, Paris, France (IUF).

* These authors contributed equally

§ These authors jointly supervised this work

¥ Present address: Institute of Neuroscience and Physiology, Department of Neurochemistry and Psychiatry, University of Gothenburg, Su Sahlgrenska, 41345 Göteborg, Sweden.

Corresponding author: Dr. Martin Giurfa

Research Centre on Animal Cognition, CNRS – UPS, 31062 Toulouse cedex 9, France

martin.giurfa@univ-tlse3.fr

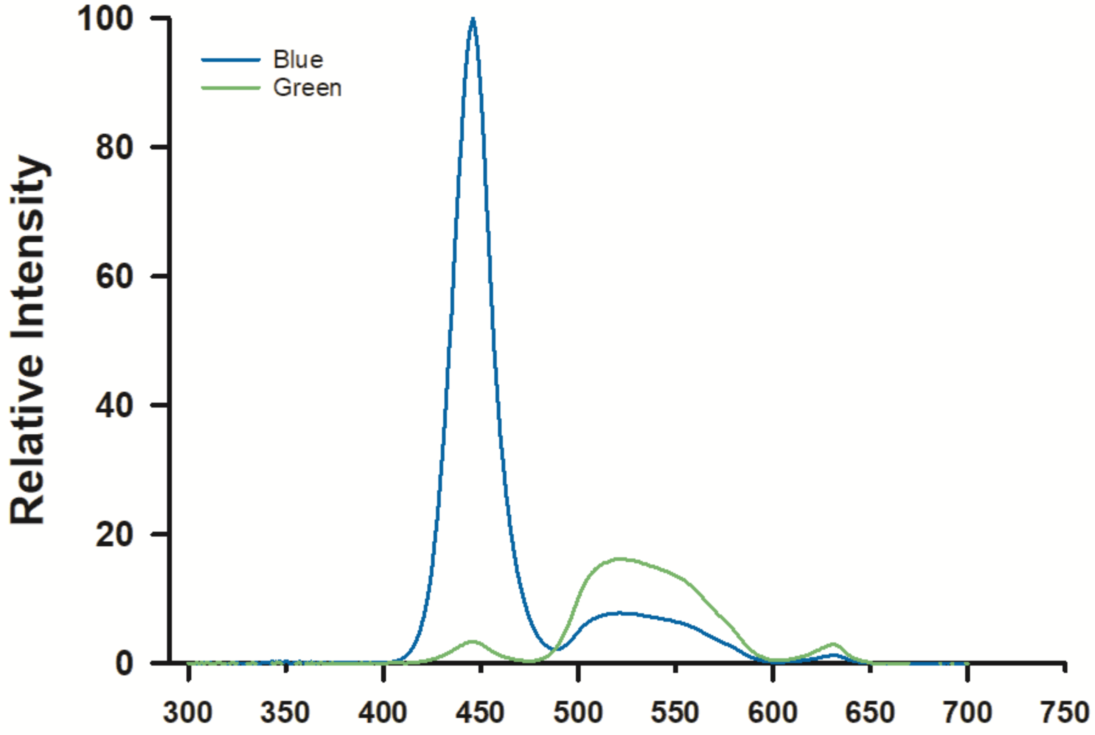

Supplementary Figure 1. Spectral distribution (relative intensity as a function of wavelength) of the blue light (dominant wavelength 446 nm) and the green light (dominant wavelength 528 nm) used to train the bees in the color discrimination task.

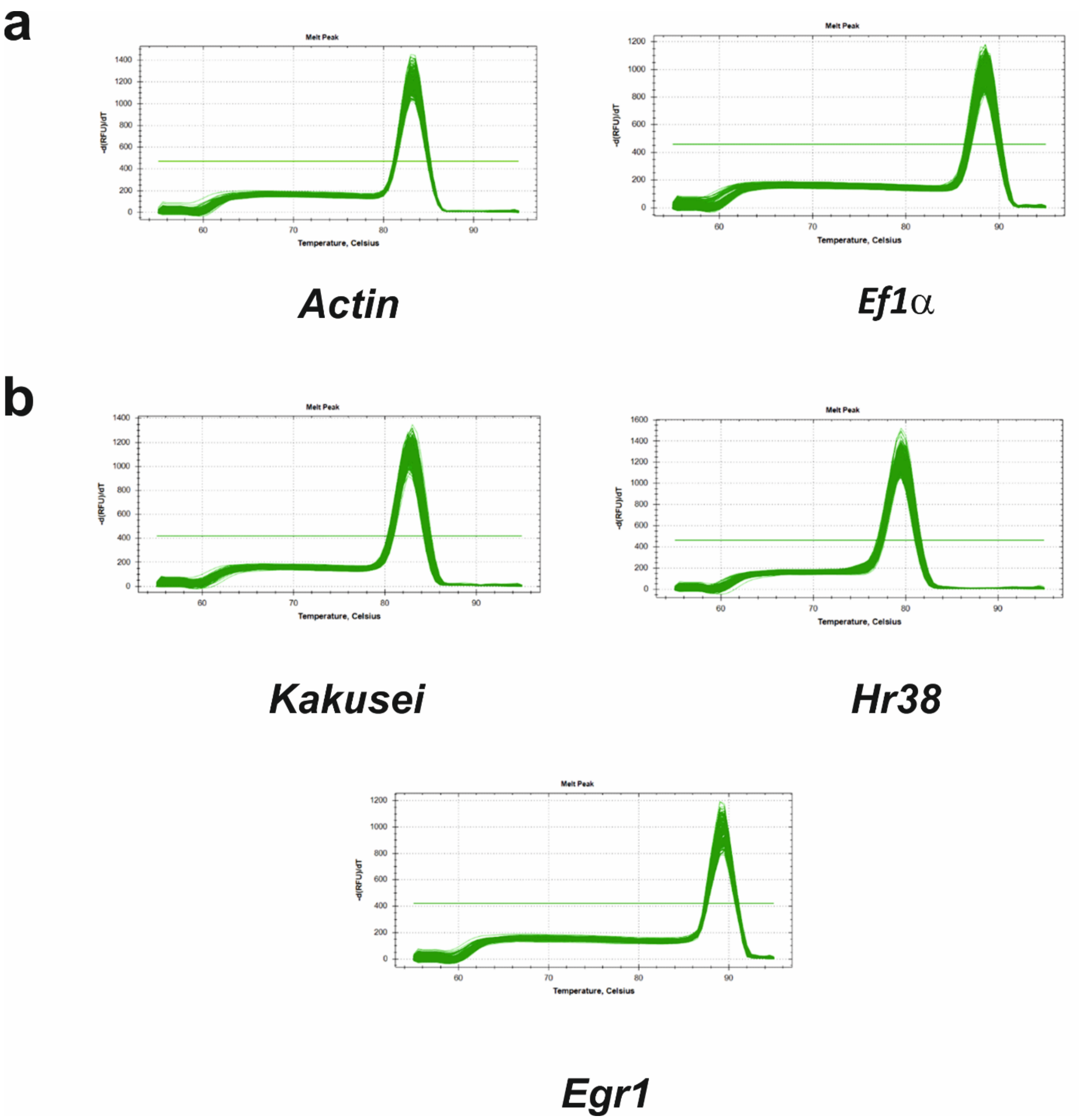

Supplementary Figure 2. Validation selectivity of gene-specific primers. Melting peaks of qRT-PCR. **a)** Reference genes. **b)** Target genes.

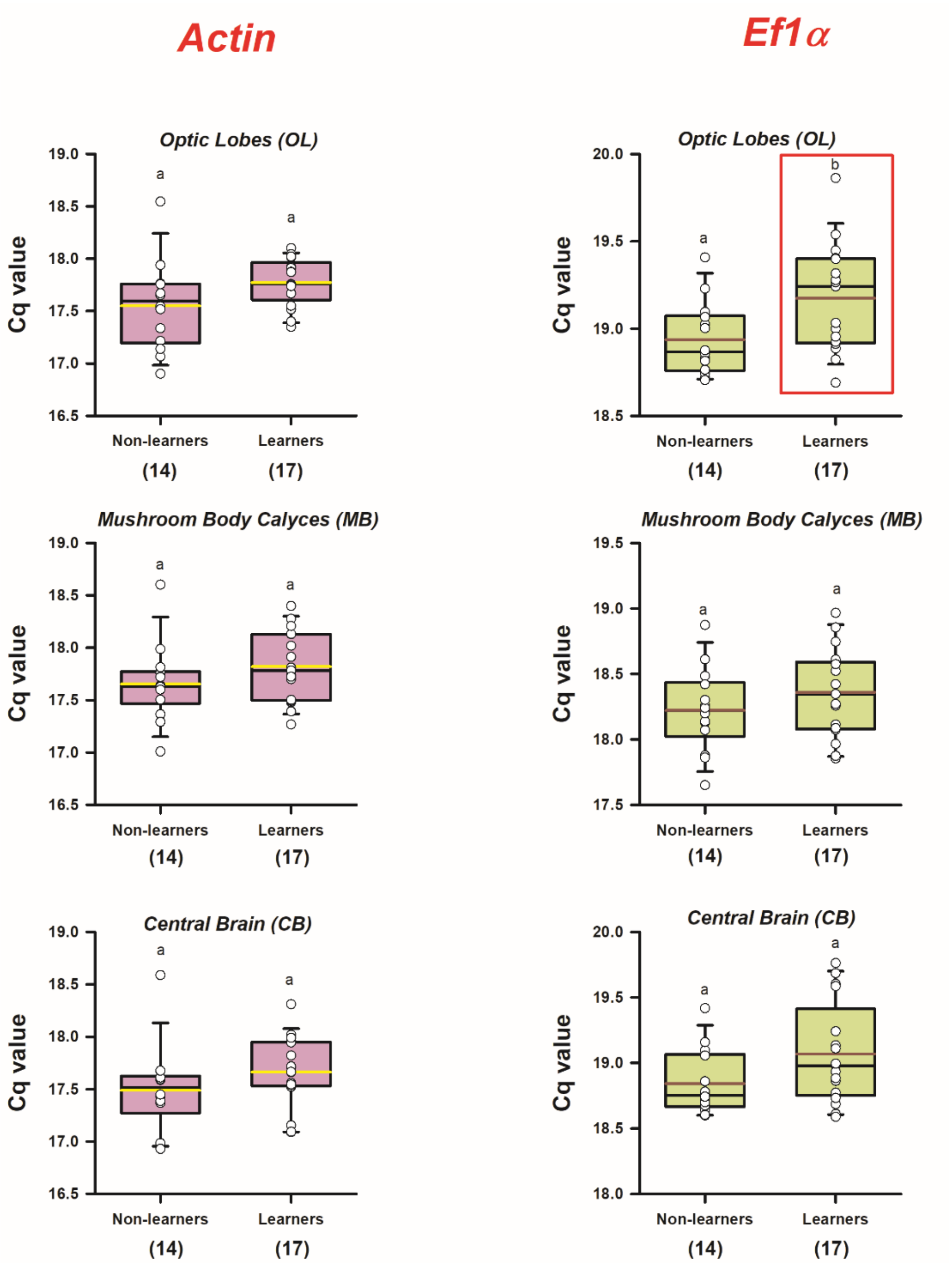

Supplementary Figure 3. Expression levels (Cq values) of the reference genes *Actin* and *Ef1α*. Expression levels are reported for the experimental groups (*Learners* and *Non-learners*) and the brain regions considered (optic lobes, mushroom body calyces and central brain). The range of ordinates was varied between graphs to facilitate appreciation of data scatter. Box plots show the mean value in yellow (*Actin*) or red (*Ef1α*). Sample sizes are indicated within parentheses below each group. Error bars define the 10th and 90th percentiles. Red boxes indicate cases in which significant variations were detected. These cases were excluded in the subsequent target analyses. For instance, for the optic lobes only *Actin* was used as reference gene, while the two reference genes were used for the other brain regions. Different letters on top of box plots indicate significate differences (two-sample t test; $p < 0.05$).
