# Supplementary material for: Visual learning in a virtual reality environment upregulates immediate early gene expression in the mushroom bodies of honey bees
Source: Commun Biol. 2022 Feb 14;5:130. doi: 10.1038/s42003-022-03075-8 (PMC8844430; doi:10.1038/s42003-022-03075-8)
Supplement: Supplementary file 2 — Reporting Summary [file 42003_2022_3075_MOESM2_ESM.pdf]

nature portfolio

Corresponding author(s): Martin GIURFA

Last updated by author(s): Dec 21, 2021

Reporting Summary

Nature Portfolio wishes to improve the reproducibility of the work that we publish. This form provides structure for consistency and transparency in reporting. For further information on Nature Portfolio policies, see our Editorial Policies and the Editorial Policy Checklist.

Statistics

For all statistical analyses, confirm that the following items are present in the figure legend, table legend, main text, or Methods section.

n/a	Confirmed	
☐	☒	The exact sample size (*n*) for each experimental group/condition, given as a discrete number and unit of measurement
☐	☒	A statement on whether measurements were taken from distinct samples or whether the same sample was measured repeatedly
☐	☒	The statistical test(s) used AND whether they are one- or two-sided<br>*Only common tests should be described solely by name; describe more complex techniques in the Methods section.*
☒	☐	A description of all covariates tested
☐	☒	A description of any assumptions or corrections, such as tests of normality and adjustment for multiple comparisons
☐	☒	A full description of the statistical parameters including central tendency (e.g. means) or other basic estimates (e.g. regression coefficient) AND variation (e.g. standard deviation) or associated estimates of uncertainty (e.g. confidence intervals)
☐	☒	For null hypothesis testing, the test statistic (e.g. *F*, *t*, *r*) with confidence intervals, effect sizes, degrees of freedom and *P* value noted<br>*Give P values as exact values whenever suitable.*
☒	☐	For Bayesian analysis, information on the choice of priors and Markov chain Monte Carlo settings
☒	☐	For hierarchical and complex designs, identification of the appropriate level for tests and full reporting of outcomes
☒	☐	Estimates of effect sizes (e.g. Cohen's *d*, Pearson's *r*), indicating how they were calculated

Our web collection on statistics for biologists contains articles on many of the points above.

Software and code

Policy information about availability of computer code

Data collection	The software used to generate the virtual-reality conditions was a custom software created by means of the Unity engine (version 2018.3.11f1). The open-source code is available at https://github.com/G-Lafon/BeeVR.
Data analysis	Statistical analyses were performed using with R 3.5.1 and Statistica 13 (TIBCO® Data Science).

For manuscripts utilizing custom algorithms or software that are central to the research but not yet described in published literature, software must be made available to editors and reviewers. We strongly encourage code deposition in a community repository (e.g. GitHub). See the Nature Portfolio guidelines for submitting code & software for further information.

Data

Policy information about availability of data

All manuscripts must include a data availability statement. This statement should provide the following information, where applicable:

- Accession codes, unique identifiers, or web links for publicly available datasets
- A description of any restrictions on data availability
- For clinical datasets or third party data, please ensure that the statement adheres to our policy

Provide your data availability statement here.

Field-specific reporting

Please select the one below that is the best fit for your research. If you are not sure, read the appropriate sections before making your selection.

☒ Life sciences ☐ Behavioural & social sciences ☐ Ecological, evolutionary & environmental sciences

For a reference copy of the document with all sections, see nature.com/documents/nr-reporting-summary-flat.pdf

Life sciences study design

All studies must disclose on these points even when the disclosure is negative.

Sample size	Sample sizes were chosen based on a previous work on visual learning in 3D VR (see Lafon et al Sci Rep 2021) in which significant color discrimination was observed under similar background conditions.
Data exclusions	Bees that were unable to choose a stimulus (i.e. that did not fulfill the criterion of a choice defined in our Method section) in at least 5 of the 10 trials used for conditioning were excluded from the analysis. In the molecular analyses, 4 non-learners brains were lost during the dissection process. Thus, the sample sizes of the non-learners differ between the behavioral (n=18) and the molecular analyses (n=14).
Replication	Behavioral data were replicable and reproduced behavioral performances already observed in a previous work on visual learning in 3D VR (see Lafon et al Sci Rep 2021)
Randomization	Bees were allocated randomly to the color contingency training (i.e. Blue Rewarded vs Green Punished or Blue Punished vs. Green Rewarded).
Blinding	Investigators performing the behavioral (GL) and the molecular analyses (HG) were different. They were blind to each other's results

Reporting for specific materials, systems and methods

We require information from authors about some types of materials, experimental systems and methods used in many studies. Here, indicate whether each material, system or method listed is relevant to your study. If you are not sure if a list item applies to your research, read the appropriate section before selecting a response.

Materials & experimental systems

n/a	Involved in the study	
☒	☐	Antibodies
☒	☐	Eukaryotic cell lines
☒	☐	Palaeontology and archaeology
☐	☒	Animals and other organisms
☒	☐	Human research participants
☒	☐	Clinical data
☒	☐	Dual use research of concern

Methods

n/a	Involved in the study	
☒	☐	ChIP-seq
☒	☐	Flow cytometry
☒	☐	MRI-based neuroimaging

Animals and other organisms

Policy information about studies involving animals; ARRIVE guidelines recommended for reporting animal research

Laboratory animals	Honey Bees Apis mellifera
Wild animals	N.A.
Field-collected samples	Honey bees were obtained from hives located in our university apiary. They were foragers captured at a feeder filled with sucrose solution to which they were previously trained.
Ethics oversight	No ethical guidance or approval is required for experimental research on honey bees.

Note that full information on the approval of the study protocol must also be provided in the manuscript.
